# Characterization of Microcirculation in Multiple Sclerosis Lesions by Dynamic Texture Parameter Analysis (DTPA)

**DOI:** 10.1371/journal.pone.0067610

**Published:** 2013-07-16

**Authors:** Rajeev Kumar Verma, Johannes Slotboom, Mirjam Rahel Heldner, Frauke Kellner-Weldon, Raimund Kottke, Christoph Ozdoba, Christian Weisstanner, Christian Philipp Kamm, Roland Wiest

**Affiliations:** 1 Support Center for Advanced Neuroimaging, University Institute of Diagnostic and Interventional Neuroradiology, Inselspital, University of Berne, Bern, Switzerland; 2 Department of Neurology, Inselspital, Bern University Hospital, and University of Bern, Bern, Switzerland; University of Regensburg, Germany

## Abstract

**Objective:**

Texture analysis is an alternative method to quantitatively assess MR-images. In this study, we introduce dynamic texture parameter analysis (DTPA), a novel technique to investigate the temporal evolution of texture parameters using dynamic susceptibility contrast enhanced (DSCE) imaging. Here, we aim to introduce the method and its application on enhancing lesions (EL), non-enhancing lesions (NEL) and normal appearing white matter (NAWM) in multiple sclerosis (MS).

**Methods:**

We investigated 18 patients with MS and clinical isolated syndrome (CIS), according to the 2010 McDonald's criteria using DSCE imaging at different field strengths (1.5 and 3 Tesla). Tissues of interest (TOIs) were defined within 27 EL, 29 NEL and 37 NAWM areas after normalization and eight histogram-based texture parameter maps (TPMs) were computed. TPMs quantify the heterogeneity of the TOI. For every TOI, the average, variance, skewness, kurtosis and variance-of-the-variance statistical parameters were calculated. These TOI parameters were further analyzed using one-way ANOVA followed by multiple Wilcoxon sum rank testing corrected for multiple comparisons.

**Results:**

Tissue- and time-dependent differences were observed in the dynamics of computed texture parameters. Sixteen parameters discriminated between EL, NEL and NAWM (pAVG = 0.0005). Significant differences in the DTPA texture maps were found during inflow (52 parameters), outflow (40 parameters) and reperfusion (62 parameters). The strongest discriminators among the TPMs were observed in the variance-related parameters, while skewness and kurtosis TPMs were in general less sensitive to detect differences between the tissues.

**Conclusion:**

DTPA of DSCE image time series revealed characteristic time responses for ELs, NELs and NAWM. This may be further used for a refined quantitative grading of MS lesions during their evolution from acute to chronic state. DTPA discriminates lesions beyond features of enhancement or T2-hypersignal, on a numeric scale allowing for a more subtle grading of MS-lesions.

## Introduction

The search for novel imaging biomarkers in multiple sclerosis (MS) has modified the concepts of neuroimaging from identifying imaging sequelae of demyelination on conventional MR-images towards strategies capable of examining functionality and pathophysiology of the MS brain. A recent technique that contributed to a better understanding of vascular changes in MS, is dynamic susceptibility contrast enhanced (DSCE-) MRI. DSCE-MRII identifies various patterns of impaired perfusion, either in non-enhancing MS lesions or NAWM, compared to healthy controls. On the other hand, inflammatory activity is accompanied by increased perfusion in lesions during the acute phase of the disease, compared to NAWM [1]–[3].

Local perfusion changes in MS lesions are currently interpreted as a consequence of local inflammation-mediated vasodilatation – a phenomenon secondary to hyperemia and blood congestion within the brain parenchyma [4], [5]. However, different observations in perfusion imaging studies in MS have challenged the interpretation of abnormal perfusion as a reactive phenomenon to inflammation. The occurrence of demyelinating lesions is not inevitably coupled to the presence of a local preceding inflammatory reaction [6], [7], and diffuse NAWM changes in the absence of structural lesions may be the consequence of a down-regulation in cerebral micro-circulation due to astrocyte dysfunction, or secondary axonal damage in the NAWM [8]. Recently, some authors suggested a formation of new outflow routes along plaque formation bypassing obstructed pathways as the reason for local perfusion changes [9].

The contribution of vascular changes to the generation of MS lesions in MS is still a matter of debate. Currently, it remains to be elucidated whether alteration in CNS perfusion in MS is a cause or rather a consequence of disease pathogenesis. From a methodological point of view, current concepts of perfusion imaging have mainly addressed vascular changes that are described either by the time needed for a contrast agent to pass the vasculature (MTT), the total volume of blood within the cerebral vasculature during the passage of a contrast bolus (CBV), or the amount of blood that perfuses the brain per time unit (CBF) [10]. Calculation of CBV, CBF, and MTT from concentration-time curves is based on the indicator dilution methods for non diffusible tracers [11]. While this technique has been clinically applied for more than a decade to investigate perfusion deficits in acute stroke [12] and altered microcirculation in brain tumors [13], [14], its application in MS is still new and MS-related perfusion characteristics has not yet been evaluated. A principal limitation of model-based perfusion imaging in MS is the variable blood-brain barrier disruption in active MS lesions requiring complex pharmacokinetic modeling to correct for extravasation of contrast medium from the plasma to the extracellular space.

A potential alternative to leakage modeling is the analysis of the tissue of interest by means of texture analysis of the MR-images at different time points along with the bolus passage of Gadolinium. Texture parameters [15]–[17] enable quantitative analyses of MR-images. Although no strict definition of image textures exists, they are described as complex visual patterns which are composed of spatially organized, repeated fingerprints characterized by individual brightness, size, shape, etc. [18]. In recent years, software developments facilitated the study of texture parameters of medical images, e.g. the MaZda package [19], [20]. Texture analysis has been applied for structural imaging in MS [21], [22] in breast cancer, liver cirrhosis, brain tumors, epilepsy or acute ischemic stroke [23]–[25]. Kassner et al. demonstrated that statistical or spectral textural features outperformed visual assessment in discriminating between tumors, as well as in discerning subtle anatomic changes associated with a high risk of seizures in patients with epilepsy [23]–[25].

Recently a novel approach has been proposed, namely Dynamic Histogram Analysis (DHA) where a simple form of texture analysis is applied to a time series of DSCE-MRII-images [14]. DHA investigates the time dependency of mean and standard deviation parameters of the voxel intensity values of the tissue of interest. This DHA approach has been successfully applied to differentiate cerebral gliomas in accordance with their histological classification [14]. In this study, a further extension to DHA is proposed, the DTPA. DTPA investigates MR difference-images, calculated by subtraction of the first steady state baseline image from images during bolus passage and reperfusion. In this paper, we extend the focus of texture analysis to quantitative studies of changes in micro-structural perfusion and leakage. This explorative study aims for investigating the differences in dynamical texture parameters between NELs, ELs, and NAWM during contrast agent passage. We hypothesized that: (a) texture parameters show characteristic tissue- and time-dependent (dynamic) behavior of MS plaques during bolus passage that differ from NAWM; and (b) statistically significant differences in texture parameters can be detected between NAWM, ELs and NELs, sufficient to discriminate the three tissue types from each other.

## Methods

### 2.1. Patients

Patients with clinical isolated syndrom (CIS) or MS diagnosed according to the 2010 McDonald's criteria, with or without immunomodulatory therapy, were included if at least one EL was present on MRI [26]. In equivocal cases, the inflammatory/demyelinating origin of the disease had to be demonstrated via biopsy and/or MR spectroscopy. Besides the clinical evaluation and MR imaging, lumbar puncture and blood tests were performed in all patients to exclude alternative diagnoses. Main exclusion criteria were any other diseases that could better explain the patient's symptoms and signs. Patients gave written informed consent prior to the study entry and the study was approved by the local cantonal ethics committee Bern, Switzerland.

### 2.2. MRI parameters

DSCE- imaging was performed on our institutes' Siemens MR-scanners using standard echo planar imaging (EPI) sequences at 1.5 T and 3.0 T. For our 1.5T scanner a TE of 47 ms and a TR of 1440 ms was used. For our 3T scanner we had a TE of 32 ms and a TR of 1500 msec. At both field strengths a FOV of 230 mm×230 mm was used with a slice thickness of 5 mm. A total of 19 parallel images with a time resolution of 1500 ms were acquired on our 3.0T system, and a total of 12 parallel images with a time resolution of 1440 ms were acquired on our 1.5T scanner. At 3.0T a total of 80 different time points were acquired, whereas on 1.5T a total of 40 different time points were sampled after bolus application. All patients received Gadobutrol (Gadovist™) 0.1 ml kg^–1^ bodyweight. Patients were positioned comfortably in the head coil and padding on either side of the head was used to help immobilization. Further the intravenous line with a long tube was put before acquisition to avoid unnecessary MRI table moving during examination.

### 2.3. Image Processing

**Figure 1:**
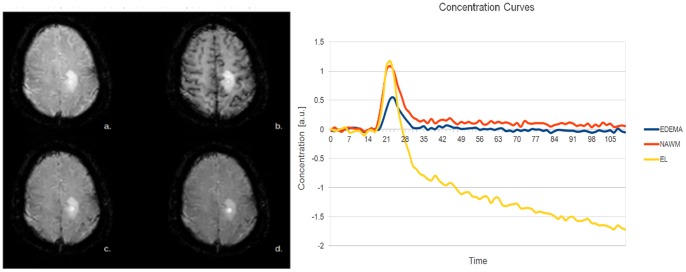
Exemplary EPI MR-images of a RRMS-patient. Data recorded: (a) at baseline; (b) at perfusion maximum; (c) at early-reperfusion, and (d) at late-reperfusion. The MS lesion shows a non-uniform structure that varies during bolus passage; (e) Concentration curves were computed within 3 TOIs; the first within the brightest circular part of the MS-lesion, the second TOI in the edema surrounding the enhancing part of the lesion; the third TOI in the NAWM on the contralateral site. The concentration curve of the EL reaches its maximum slightly earlier in time than the NAWM due to BBB disruption. Interestingly, the contrast time curve of edema reaches less high concentrations, indicating hypoperfusion of the edema.

The image analysis method we applied focuses on DSCE-*difference* images: These difference images are computed from the difference between the first steady state EPI image and images recorded during bolus arrival, passage and reperfusion. In mathematical terms, let 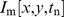 
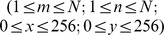
be the 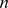-th image of the image time series at slice location 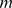. The difference images 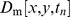 are computed as follows: 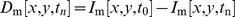. In case of a non-leaky blood brain barrier (BBB), the contrast of 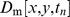 is such that the brighter the voxel of 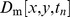, the more contrast agent there is in the voxel at that time point 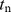. From 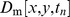 all time dependent texture parameter maps (TPMs) are computed, resulting in 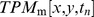. The rationale for the computed texture maps in this study is given in section “Computed TPM types during BLP, IFP, OFP and RFP” below. To illustrate the time-dependent perfusion behaviour in a EL, figure 1 a–d display typical DSCE EPI images during the four time-intervals that are investigated in this study.

#### 2.3.1. Analysis of texture parameter maps

**Figure 2:**
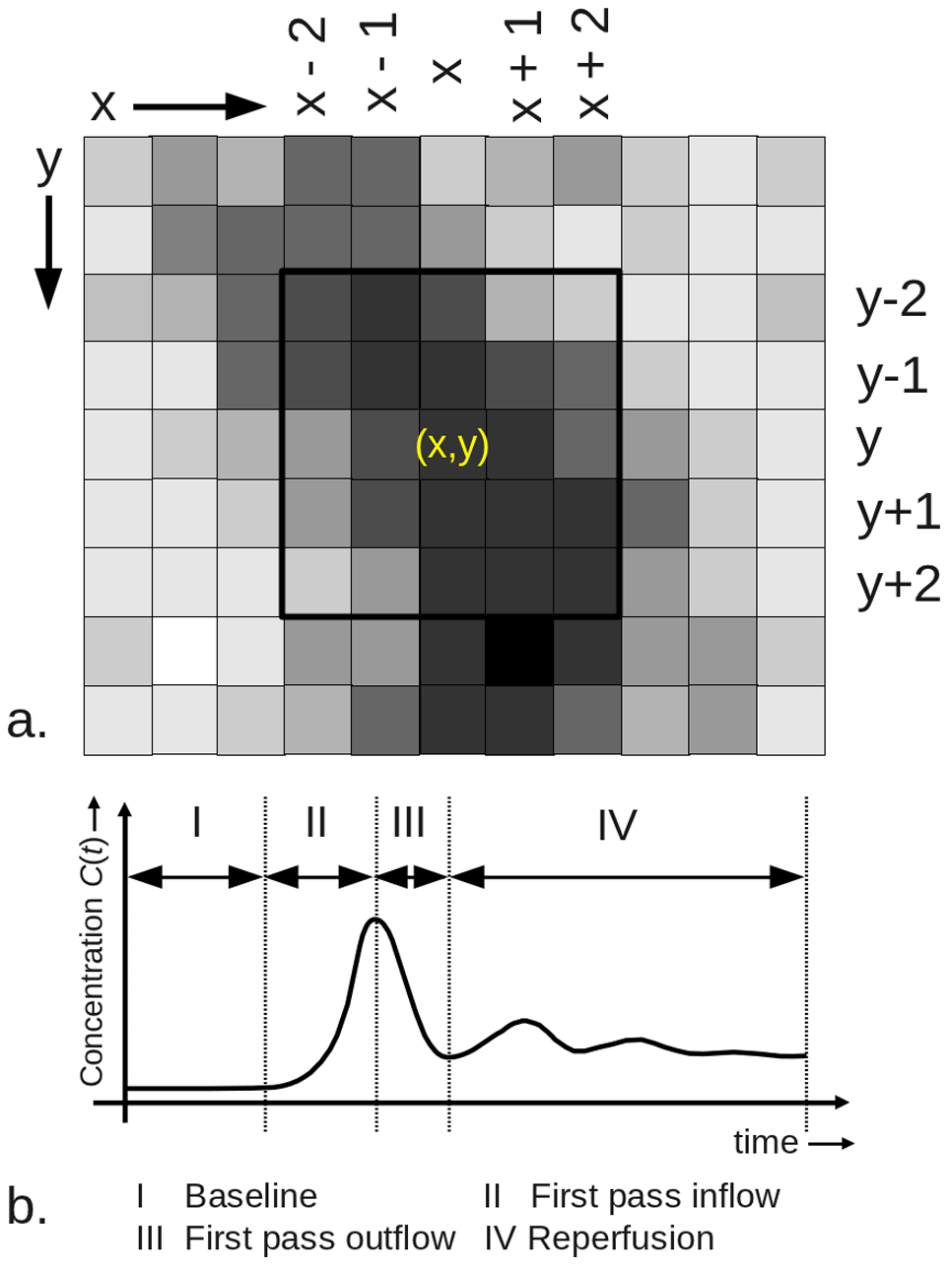
a. 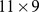 voxel region of an MR-image. To a simple moving average filter, the developed algorithm computes for each voxel 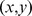 of the image time series 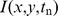, where 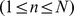, the local (statistical) texture parameters based on a user definable input voxel area (in this case 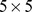). b. Schematic contrast agent time curve graphically indicating the definitions of the time-intervals over which the image characteristics are averaged, discriminating the baseline period (I), inflow period (II), outflow period (III), and reperfusion period (IV).

**Figure 3:**
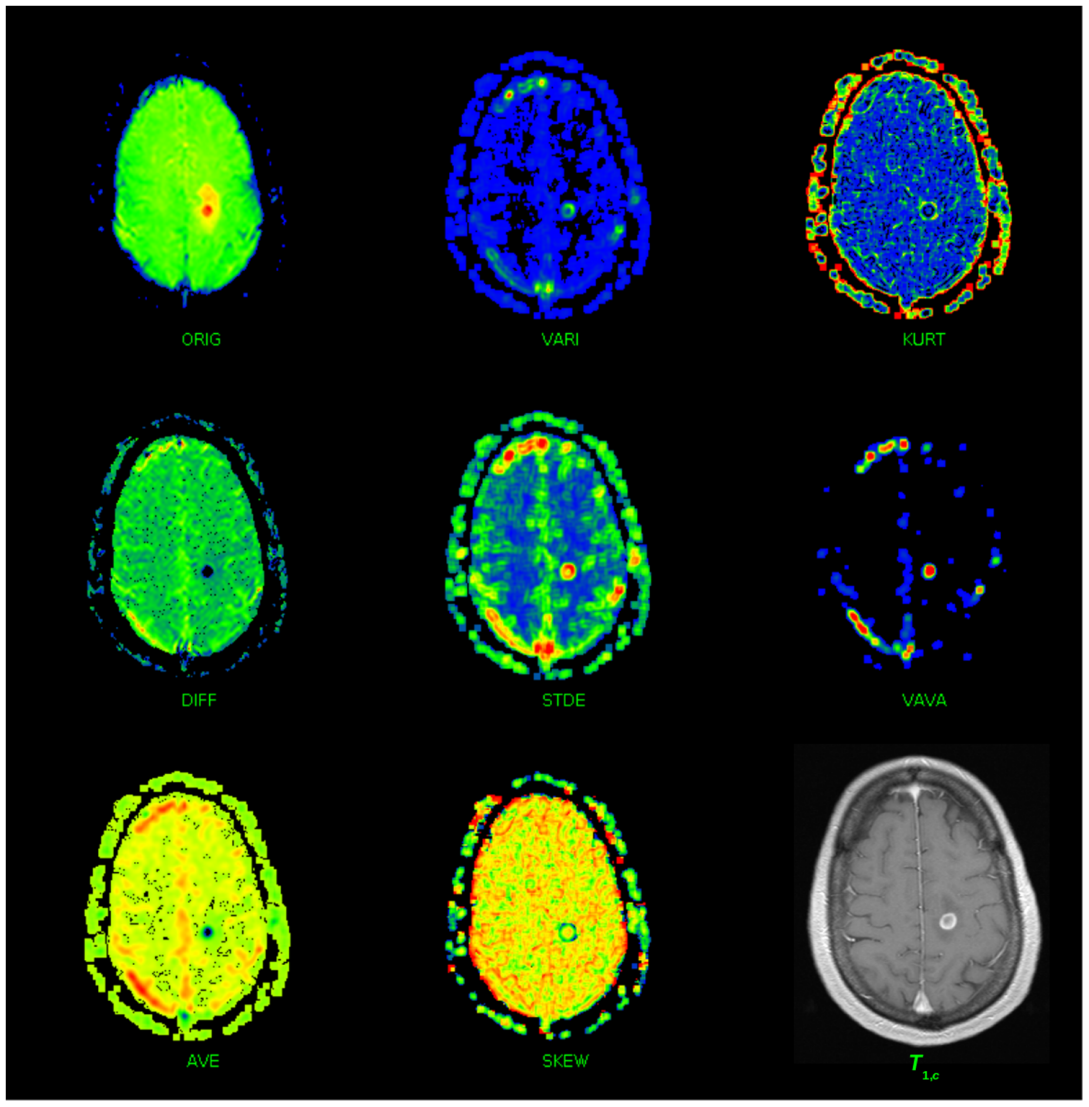
Texture maps computed from the original EPI MR-image time series (ORIG). Data recorded at *one* time point during the RPP during DSCE imaging. The texture map DIFF time series indicates the difference maps of the TPM-ORIG time series; TPM-AVE indicates the local average map; TPM-VAR indicates the local variance map; TPM-STDEV indicates the local standard deviation map; TPM-SKEW indicates the local skewness parameter map; TPM-KURT indicates the local kurtosis map; and TPM-VAVA indicates the local variance-of-variance map of the TPM-DIFF time series. The displayed maps are function of time and only one map is displayed here at a time point during which signal enhancement due to extravasation of contrast agent already took place. Note that the lesion area that shows enhancement (best seen as isolated red dot in the TPM-VAVA) is much smaller than the total lesion (red, orange and yellow parts) in the TPM-ORIG. Additionally, the lower right image is T1w after application of contrast agent.

Figure 2 shows schematically a 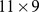 voxel region detail of an MR-image 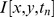 at a time point 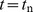 during bolus passage. Each voxel 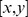 is assigned with a specific voxel intensity distribution; e.g. a voxel 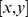 in the center of NAWM will have a different voxel intensity distribution compared to a voxel 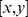 centered in the gray matter (see Figure 1b). Gray matter, due to its strong vascularisation, will in general be more hypointense during in-, out- and reperfusion-phases compared to voxels within NAWM. Furthermore, NAWM constitutes a smaller range of voxel intensity distribution than voxels closer to the interface between two tissue types. This results in a highly variable voxel intensity distribution. The statistical properties of regions with variable center points 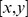 strongly differ within an image as a function of tissue type, and of the position within a tissue (e.g. representing a voxel at the periphery or in the center of the tissue). Extending our previous work [14], we now incorporated further histogram based texture parameters in the analysis of voxel intensity distributions computed from the DSCE-EPI images. Texture parameters computed as a function of the location 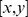 can be used to characterize these (non-) Gaussian voxel intensity distributions. In order to quantify these textures as a function of position 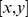 and time, we developed an application that is called dynamic texture parameter analyzer (DTPA). This application computes a series of different texture parameters for user-defined square voxel neighbour kernel size; in this paper we used a 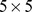 voxel range (Figure 2), for every region around 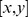 as a function of time. In this manner, a time series of texture parameter images or maps (TPMs) is obtained. For illustration, an example of the TPMs computed by the application at one time point during the reperfusion phase is given in Figure 3. In this paper we focused on the first four moments of the mean and their variances during baseline, contrast agent inflow, outflow, and reperfusion phase to characterize MS-lesions of different types (NEL, EL), and NAWM.

#### 2.3.2. Data Normalization

Our study sample has been recorded on three MR scanners runnung at with 1.5 and 3T of the same vendor using the same DSCE-Perfusion sequence. Differences in field strength, patient head size, and head positioning lead to significant differences in RF-coil-load and thus to changes in MRI-voxel intensities 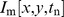 from patient to patient. Additionally, in DSCE-MRI the voxel intensities of the difference image series 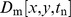 do not only depend on the flow properties of the blood vessels, but also on the cardiac ejection fraction. In order to be able to compare the texture parameters between two different patients, normalization for both sources of variance is mandatory. Since both 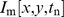 and 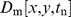 derived texture parameters are studied, a twofold normalization is required:

#### 2.3.3. Corrections for Differences in RF-coil-loads

Normalization for these differences was performed by setting a user defined region encircling a specific tissue of interest (TOI) to a certain value. The normalization constant 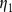
is defined as the quotient of average voxel intensity value of voxels within this user-defined region 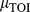 divided by a user-defined constant 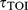, that resembles the ideal value for this tissue type, so 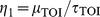, and the normalized image time series 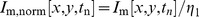 for all 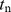. With the selection of the most homogeneous tissue located at a predefined anatomic structure, the inter-subject variance can be minimized further. In this study, normalization was performed by defining a region in NAWM of the first baseline image and with a 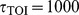.

#### 2.3.4. Correction for different cardiac ejection fractions

Tissue concentrations 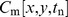 time curves (see Figure 2b) as a function of time can be computed from the formula 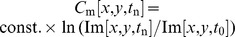. Depending on the cardiac ejection fraction, the duration of inflow and outflow phase may differ substantially, resulting in different peak contrast concentration values that prevent a time-point-by-time-point comparison between patients for 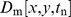 and all its derived time dependent texture parameters. To overcome this problem, we compared identical time intervals of normalized 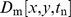 as described in [14]. The following time intervals were defined (see also Figure 2b):

Base line period (BLP): time interval between the first measure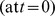, and time of arrival (TOA) at 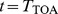.
Inflow period (IFP): time interval between 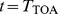 and 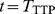.
Outflow period (OFP): time interval 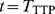 and 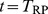 (time point where the concentration reaches its first local minimum after 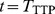).
Reperfusion period (RPP): time interval between 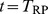
and 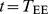, (here 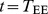 is time at the end of the measurement).

For the normalization of inter-patient differences due to differences in cardiac ejection fraction the area-under-the-time-difference-curve 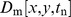 of a user defined TOI is determined for the IFP and OFP and is denoted by 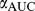. Within the scaling factor 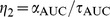 the dominator 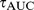 is set to 200, for all images 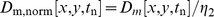. In the present implementation, 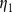 and 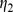 are determined for the same region in NAWM.

The time points demarking the four time intervals (BLP, IFP, OFP, RPP) are determined automatically by the developed program by numerical analysis of the time dependent bolus passage function. Due to differences in timing caused e.g. by differences in cardiac function, only separately time averaged values over the four defined time intervals as defined above (BLP, IFP. OFP, RPP) can be adequately compared.

#### 2.3.5. Computed TPM types during BLP, IFP, OFP and RPP

This section shortly describes the computed histogram based on TPMs. Figure 3 illustrates all derived texture parameter maps computed in this study for a single patient with RRMS.

1. Original EPI MR image time series: TPM-ORIG The original 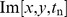 EPI image series can be regarded as a texture map itself, since every type of MR-pulse sequences is designed to obtain a specific image contrast, i.e. having specific textural characteristics. DSCE-raw images show limited but substantial contrast differences during the BLP, so that both EL and NEL can clearly be identified (Figure 1a). In case of an intact BBB, the passage of Gadubutrol causes 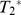-shortening effects resulting in a signal decrease relative to the BLP. Counteracting this 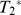-shortening effect, voxel intensities 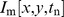 *increase* during the RPP and OFP in ELs with a disrupted BBB. The leakage effect during the OFP and RPP is illustrated in Figure 3, (denoted as “ORIG”). The EL (red) can be clearly depicted.

2. Difference image time series: TPM-DIFF The brightness of a voxel is proportional to its Gadobutrol concentration for the difference image series 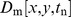 if the BBB is preserved. In case of BBB disruption 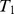-shortening effects in the tissue result in a signal *decrease* during the first passage of the contrast agent and reperfusion (see Figure 1e and Figure 3 DIFF). This TPM is further denoted by TPM-DIFF.

3. Local Average Difference Maps: TPM-AVER This TPM, denoted by 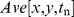, is the moving average filtered version of TPM-DIFF over a 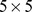 voxel intensity range. In Figure 3, the TPM denoted with “AVE” gives an example of the appearance of this texture, illustrating better SNR, but worse spatial resolution. This TPM was added to provide the same spatial resolution as the other textures, and is denoted by TPM-AVER.

4. Local Variance: TPM-VARI The TPM-VARI 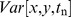 time series computed from 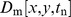 is sensitive to edges within the image. The broader the local voxel intensity range, the brighter it appears in this TPM. To some extent this texture is related to the gradient image of 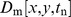. This texture thus reflects the *local heterogeneity* of a tissue [14]. In Figure 3, the texture map indicated with “VARI” is an example of this texture type, illustrating how an EL appears in this type of TPM. Note that the surrounding edema behaves differently. This TPM is further denoted by TPM-VARI.

5. Local standard deviation: TPM-STDEV The TPM 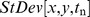 is derived from 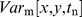 by taking its square root. This TPM is further denoted by TPM-STDEV.

6. Local Skewness: TPM-SKEW 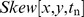 is the third moment about the mean of 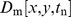 (see Figure 3, “SKEW”). This TPM-SKEW is a measure of the *asymmetry* of the voxel intensity distribution, and in this case indicates the asymmetry of contrast agent distribution within the image voxels. An increase in asymmetry reflects an increase in skewness. For positive skewness, there is a preference in the distribution for brighter voxel intensity values, and for a negative skewness there is a preference for darker voxel intensity values.

7. Local Kurtosis: TPM-KURT 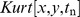 is the fourth moment about the mean of 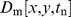 (see Figure 3, “KURT”). This texture parameter measures the steepness or peakedness of voxel intensity distribution. The software computes the excess kurtosis, which sets the kurtosis for Gaussian distributed voxel intensities to zero. Voxel intensity distributions which are less peaked than the Gaussian distribution have an excess 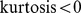 (platykurtic), and voxel distributions more peaked than the Gaussian have an excess 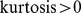 (leptokurtic). This kurtosis TPM is denoted by TPM-KURT.

8. Local variance-of-variance: TPM-VAVA 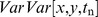 of 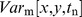 is the variance-of-the-variance TPM, indicated as TPM-VAVA. Since 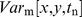 is a stochastic variable itself, it also has a variance (see Figure 3, “VAVA”). Due to the large dynamic range of the TPM-VAVA and the limitation to 512 color discretization display-levels in the current implementation (see Figure 3), low intensity voxel values are all displayed in black, although they all have distinct floating point values. Interestingly, in contrast to the TPM-VARI this texture is not an “edge detector” as can be clearly seen in the image.

#### 2.3.6. Determined Texture Parameters

**Figure 4:**
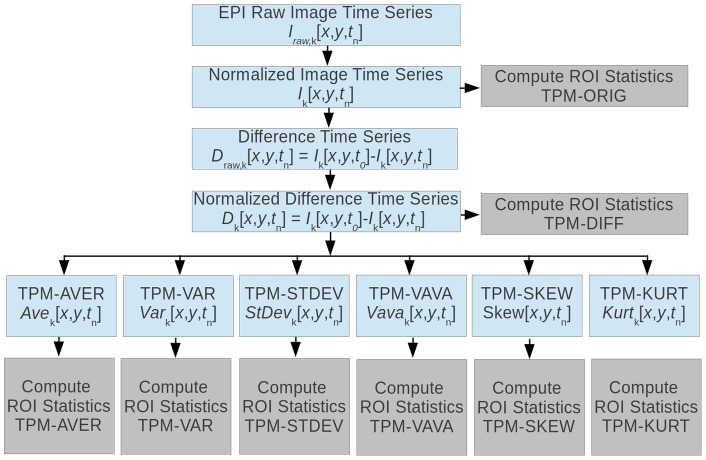
Relation between the original EPI raw image time series (ORIG-TPM) and derived image time series. The “difference image time series” (TPM-DIFF) is obtained by subtracting the 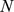-th image from the first image, resulting in an image series for which holds that, the brighter the voxel, the more contrast agent is present at that location at the specific point in time. All other histogram based TPMs are derived from the TPM-DIFF image series and are computed over the same 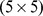 voxel-region in order to improve SNR: local average (TPM-AVE, moving averaged version of TPM-DIFF), local variance (TPM-VAR), local standard deviation (TPM-STDEV), local skewness (TPM-SKEW), local kurtosis (TPM-KURT) and local variance of variance (TPM-VAVA). Since each texture map is an image itself, the statistical parameters of a user defined TOIs can be computed for each TPM denoted by the boxes denoted with “Compute TOI statistics”. For each TPM and each TOI the following statistical parameters were computed: mean, standard deviation, variance, variance-in-variance, skewness, and kurtosis.

Within the texture maps as displayed in Figure 3, manually shaped regions can be drawn. The basic statistical properties of these regions (NAWM, EL, and NEL) are determined within all TPMs. To clarify the interdependence of the TPMs, Figure 4 displays the TPMs (eight dark gray boxes), and the parameters determined from them. For each TOI (EL, NEL or NAWN) defined in the TPMs and for each time interval, the six statistical parameters (light gray box) are determined, resulting in a total of 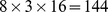 parameters. The user defines the TOIs in one of the eight TPMs and the TOIs are copied fully automatically into all other TPMs.

#### 2.3.7. Selection Criteria for the TOI

For this pilot study, all TOIs (EL, NEL and NAWM) were defined by a board certified radiologist (RV) with 12 years of experience in the diagnostic of MS lesions. The following selection criteria were maintained: (a) a contour was drawn around the complete hyperintense NEL in an image of the BLP; (b) a contour was drawn enclosing the hypointense part of the EL at the end of the RPP, i.e. in the last image of the time series; and (c) the NAWM contour was drawn in the WM at the end of the IFP, i.e. within the time period where the best GM/WM contrast was obtained. For every patient at least one image covering one or more lesions was analyzed, and a maximum of 3 EL and 3 NEL lesions was not exceeded. A total of 37 TOIs were defined in NAWM, 27 TOIs in ELs, and 29 TOIs in NELs. In most cases lesions and NAWM were analyzed on the same slice. Since in a few cases only one lesion and the corresponding NAWM were analyzed, the number of NAWM measurements exceeds the number of EL and NEL measurements. Four out of 27 ELs were surrounded by vasogenic edema (see Figure 1). Additionally to the DTPA we calculated the bolus passage (i.e. perfusion) characteristics of every TOI [27].

### 2.4. Workflow

Data analysis included the following workflow steps: (a) launch DTPA analyzer tool; (b) mount examination and load DICOM DSCE image-series (TPMs are computed and displayed); (c) draw contour in NAWM and press normalize button (data are twofold normalized now); (d) define the TOIs by drawing contours around EL, NEL and NAWM contours and press reporting button; all statistical parameters are written to text files that can be easily imported into a statistical analysis application; and (e) per TPM with three different tissue types defined, six parameters were determined for each tissue type, and for each of the four time intervals separately, making a total of 72 parameters, where 72 independent Wilcoxon-tests were performed in order to test for differences between the tissue types.

### 2.5. Statistical evaluation

The statistical software package R-Studio (0.96.331, available from http://www.rstudio.org/) was used for the statistical analysis of the data. In this explorative study we investigated (a.) which dynamic texture parameters discriminate between NAWM, NEL and EL, and (b.) during which time interval (BLP, IFP, OFP or RPP) these tissue types show most discriminative properties. The distribution of all texture parameters was examined in advance, in order to be able to select the correct test statistic. Since the texture parameters that we determined were not normally distributed, we employed a one way ANOVA followed by Wilcoxon sum rank tests. If the p-value of the ANOVAs F-test satisfied the condition p<0.05, the multiple Wilcoxon sum rank tests between NAWM, NEL and EL were performed. The p-values of these tests were corrected for multiple Wilcoxon sum rank testing by setting the “BH” option, which stands for the Benjamini, Hochberg, and Yekutieli method to control for false discovery rate. The false discovery rate of this method is a less stringent condition than the family-wise error rate, so these methods are more powerful than e.g. the Bonferroni test.

## Results

### 3.1. Clinical data

The results of every patient regarding gender, age, diagnosis, EDSS, disease duration, disease modifying treatment (DMT), acute disease exacerbation and symptomatic EL are presented in Table 1. 18 patients (83.3% female) with a mean age of 38.9 years (range 20–74 years) and a mean disease duration of 7.7 years were enrolled. 15 patients had relapsing-remitting MS (RRMS), two patients secondary progressive MS (SPMS) and one patient CIS. Three of these patients had tumefactive lesions and biopsy was performed in two of them showing predominately macrophage and T-cell mediated demyelination in one and antibody-mediated demyelination in the other patient (Table 1) corresponding to pattern I (patient 5) and II (patient 2) of the classification of demyelination by Lassmann and Lucchinetti [28]. In the third patient, MR-spetroscopy and MR-angiography was sufficient to further underline the inflammatory/demyelinating origin of the lesion(s) (patient 1). 12 patients presented with acute disease exacerbation at the time of MRI, with symptomatic ELs in eight patients. None of the patients received steroid treatment before or at the time of MRI.

**Table 1:** Details of patients included in the study.

No.	Gender	Age (years)	Diagnosis	EDSS	Disease duration	DMT-therapy	Acute disease exacerbation/start before MRI	Symptoms of acute disease exacerbation	Symptomtic EL
1	female	30	RRMS/TL	2	17 months	none	yes/4–5 days	hemihypaesthesia left	yes
2	female	64	CIS/TL	3	new onset	none	yes/7–8 days	dysarthria/ataxia	yes
3	female	54	RRMS	2	24 years	none	yes/5–6 days	paresis right leg	yes
4	female	33	RRMS	2	new onset	none	yes/2 days	sensorimotor hemisyndrom left	yes
5	female	30	RRMS/TL	3	new onset	none	yes/3 days	hemiparesis right	yes
6	male	29	RRMS	1.5	new onset	none	yes/12 hours	retrobulbar neuritis left	no
7	female	24	RRMS	2	3 months	Betaseron®/Betaferon®	no	N/A	no
8	female	37	RRMS	6.0	6 years	none	yes/9 hours	hemiparesis left, anarthria	yes
9	female	20	RRMS	1.5	27 months	Betaseron®/Betaferon®	no	N/A	no
10	female	24	RRMS	4	26 months	Copaxone®	no	N/A	no
11	female	60	SPMS	7.5	23 years	None	yes/2 days	hemiparesis left	yes
12	female	50	RRMS	3.5	23 months	Betaseron®/Betaferon®	no	N/A	no
13	female	23	RRMS	1	new onset	none	yes/2–3 months	progressive hypesthesia in all extremities	no
14	female	44	RRMS	3.5	2 months	none	yes/3 days	dysaesthesia, ataxia, bladder disorder, fatigue	yes
15	female	35	RRMS	5.0	13 years	none	yes/4 month	progressive paresis in lower extremity	no
16	female	42	RRMS	2	new onset	none	no	spasticity in all extremities	no
17	male	74	SPMS	7	30 years	none	yes/2 days	paraparesis	no
18	male	28	RRMS	1.5	7 months	Betaseron®/Betaferon®	no	N/A	no

MS  =  Multiple Sclerosis; RRMS  =  relapsing-remitting MS; SPMS  =  secondary-progressive MS, CIS  =  clinically isolated syndrome; TL  =  tumefactive lesion; EL  =  enhancing lesion; DMT  =  disease modifying treatment; N/A  =  not applicable.

### 3.2. Discriminators between two tissue types

Table 2 summarizes the number of statistical differences obtained for all TPMs as a function of the time interval and texture parameter type: For the different periods IFP, OFP and RPP we calculated the parameters, that discriminated between two tissue types (NEL vs. EL, NEL vs. NAWM or EL vs. NAWM). Measured during all timed periods, the most discriminative TPM is TPM-ORIG, followed by TPM-STDEV and TPM-VARI. Within the four preselected time periods that have been analysed, during the RPP the highest number of discriminative TPMs were detected. During RPP, for the TPM-STDEV 11 different TPs were detected that discriminated differences in the tissue under investigation. During OFP, TPM-STDEV (8 TPs) and TPM-VARI (8 TPs) were most discriminative, while during IFP, TPM-DIFF (9 TPs) yielded the most significant results.

**Table 2:** Overview of total numbers of significant texture parameters per time interval and parameter type.

Texture Type	BLP	IFP	OFP	RPP	Total
**TPM-AVER**	2	6	4	11	23
**TPM-DIFF**	2	9	6	9	26
**TPM-KURT**	2	6	0	2	10
**TPM-ORIG**	8	8	6	9	31
**TPM-SKEW**	1	0	2	2	5
**TPM-STDE**	2	7	8	11	28
**TPM-VARI**	2	8	8	9	27
**TPM-VAVA**	1	8	6	9	24
**Total**	20	52	40	62	174

Total number of performed tests are 8×72 = 576. Expected false positive rate (p = 0.05) 28.8. However, the number of observed positive Wilcoxon tests are 174, strongly indicating that observed differences in the analyzed texture maps are not just by chance.

Overall, the differences in tissue types between between EL, NEL and NAWM are predominantly expressed during RPP (62 out of 144 parameters were discriminative). The TPM types with least significant discrimination between the TOIs are the TPM-SKEW, with only (5 TPs), and TPM-KURT with 10 significant different TPs.

### 3.3. Discriminators between all TOIs

Table 3 summarizes all parameters that discriminate NAWM, NEL and EL. This table lists p-values of the One-way ANOVA (p<0.05). In addition, we performed a repeated Wilcoxon sum rank tests corrected for multiple comparisons using the Benjamini-Hochberg method. TPM-DIFF maps, TPM-STDEV and TPM-VARI were the strongest discriminators between the TOIs, and yielded the highest significance levels during the OFP, followed by the RPP. The TPM-ORIG was less discriminative between the TOIs.

**Table 3:** Overview of all texture parameters for which there exist statistical significant differences between all three TOIs.

Texture Map	Stat. par.	Time period	Anova (p-value)	NAWM vs EL (p-value)	NAWM vs NEL (p-value)	NEL vsEL (p-value)
**TPM-AVER**	st-dev	RPP	0.000114	0.0018	0.047	0.047
**TPM-AVER**	var	RPP	0.001508	0.0011	0.0357	0.0357
**TPM-AVER**	vava	RPP	5.735.10^−11^	7.7.10^−10^	0.0038	3.0.10^−5^
**TPM-DIFF**	vava	IFP	4.43.10^−5^	4.1.10^−5^	0.036	0.03
**TPM-DIFF**	st-dev	OFP	2.635.10^−5^	2.2.10^−6^	0.0235	0.0041
**TPM-DIFF**	var	OFP	0.0004425	9.4.10^−7^	0.0213	0.0033
**TPM-DIFF**	vava	RPP	2.573.10^−11^	2.1.10^−9^	0.0028	1.2.10^−5^
**TPM-ORIG**	var	RPP	8.732.10^−5^	8.6.10^−9^	1.5.10^−07^	0.049
**TPM-STDEV**	mean	IFP	0.0002377	4.4.10^−5^	0.029	0.02
**TPM-STDEV**	mean	OFP	1.403.10^-8^	1.5.10^−9^	0.0016	2.8.10^−5^
**TPM-STDEV**	vava	OFP	0.02834	2.9.10^−7^	3.1.10^−5^	0.027
**TPM-STDEV**	vava	RPP	2.5.10^−5^	1.1.10^−8^	4.10^−4^	3.559.10^−11^
**TPM-VAR**	var	IFP	0.003175	5.8.10^−5^	0.03	0.011
**TPM-VAR**	vava	IFP	2.501.10^−6^	2.9.10^−5^	0.0025	0.0161
**TPM-VAR**	mean	OFP	3.415.10^−6^	3.0.10^−9^	0.0014	6.8.10^−5^
**TPM-VAR**	st-dev	OFP	0.002716	4.110^−5^	0.038	0.0074

The significance levels of the one way ANOVA and the multiple Wilcoxon sum rank tests are indicated.

### 3.4. The Time Evolution of Texture Parameters

As an example, Table 4 displays the derived time evolution of all texture parameters for TPM-STDEV derived statistical parameters. Two perfusion imaging parameters discriminated between the different tissue types, as investigated in this study, were mean TPM (p<0.002) and TPM-VAVA (p<0.0001). The strongest effects could be denoted for the mean TPM during the outflow for the VAVA TPM during the reperfusion period (p<0.002). Notably, only the mean TPM discriminated between NAWM and EL during inflow, while all six parameters discriminated the ELs during the outflow period (p<0.05). The best discrimination between EL and NAWM was achieved by the VAVA TPM (p<3.5×10^−11^) during outflow and reperfusion. NEL were discriminated from NAWM by three parameters during outflow (mean TPM, p<0.002; TPM-SKEW, p<0.03 and TPM-VAVA (p<4×10^−4^). The best discrimination between EL and NAWM and NEL and NAWM was achieved by the TPM-VAVA during reperfusion. For discrimination between NEL and EL, the mean TPM during reperfusion was the best discriminator (p<9.7×10^−6^), followed by mean TPM during outflow (2.8×10^−5^). Notably, the TPM-VAVA contributed less to the discrimination of NEL and EL during outflow (p<0.03) and reperfusion (p<2.5×10^−5^). During the inflow period, the mean TPM was the best discriminator between NAWM and EL (4.4×10^−5^), while TPM-VAVA most reliably discriminated between NAWM and NEL (2.5×10^−5^). In summary, the mean TPM could be used as a discriminator between EL, NEL and NAWM during inflow and outflow, while TPM-VAVA discriminated during the outflow and reperfusion period. The TPM-VAR, the TPM-SKEW and TPM-KURT failed to discriminate between the different tissue types.

**Table 4:** All ANOVA test values, and Wilcoxon sum rank test results (p-values) for all statistical parameters and for all time periods of the TMP-STDEV texture parameter map.

a.) Base line period				
Parameter	ANOVA Pr(>F)	NEL vs EL	NAWM vs EL	NAWM vs NEL
**Mean**	0.2932	0.4	0.23	0.46
**St-dev**	0.8974	0.93	0.93	0.93
**Var**	0.8718	0.91	0.91	0.91
**Vava**	0.001283	0.5625	0.002	0.0037
**Skew**	na	na	na	na
**Kurt**	na	na	na	na

b.) Inflow period				
Parameter	ANOVA Pr(>F)	NEL vs EL	NAWM vs EL	NAWM vs NEL
**Mean**	0.0002377	0.02	4.4e–05	0.029
**St-dev**	0.2267	0.238	0.065	0.366
**Var**	0.4992	0.19	0.05	0.38
**Vava**	3.841.10^−6^	0.14907	2.5.10^−5^	0.00032
**Skew**	0.04121	0.962	0.046	0.016
**Kurt**	0.207	0.731	0.12	0.035

c.) Outflow period				
Parameter	ANOVA Pr(>F)	NEL vs EL	NAWM vs EL	NAWM vs NEL
**Mean**	1.403.10^−8^	2.8.10^−5^	1.5.10^−9^	0.0016
**St-dev**	0.003319	0.0614	0.0047	0.1309
**Var**	0.03201	0.0775	0.0033	0.102
**Vava**	0.02834	0.027	2.9.10^−7^	3.1.10^−5^
**Skew**	0.0736	0.637	0.024	0.026
**Kurt**	0.3643	0.423	0.045	0.127

(d.) Reperfusion period				
Parameter	ANOVA Pr(>F)	NEL vs EL	NAWM vs EL	NAWM vs NEL
**Mean**	2.813.10^−7^	9.7.10^−6^	1.1.10^−7^	0.11
**St-dev**	3.987.10^−5^	0.0074	0.0014	0.4354
**Var**	0.03201	0.0091	0.001	0.3672
**Vava**	3.559.10^−11^	2.5.10^−5^	1.1.10^−8^	4.10^−4^
**Skew**	0.03806	0.4	0.015	0.015
**Kurt**	0.1626	0.5821	0.0075	0.0041

The program did not determine the skewness and kurtosis during the base line period and are therefore indicated by “n.a”.

### 3.5. All TPMs Mean Parameters as a Function of Time

**Figure 5:**
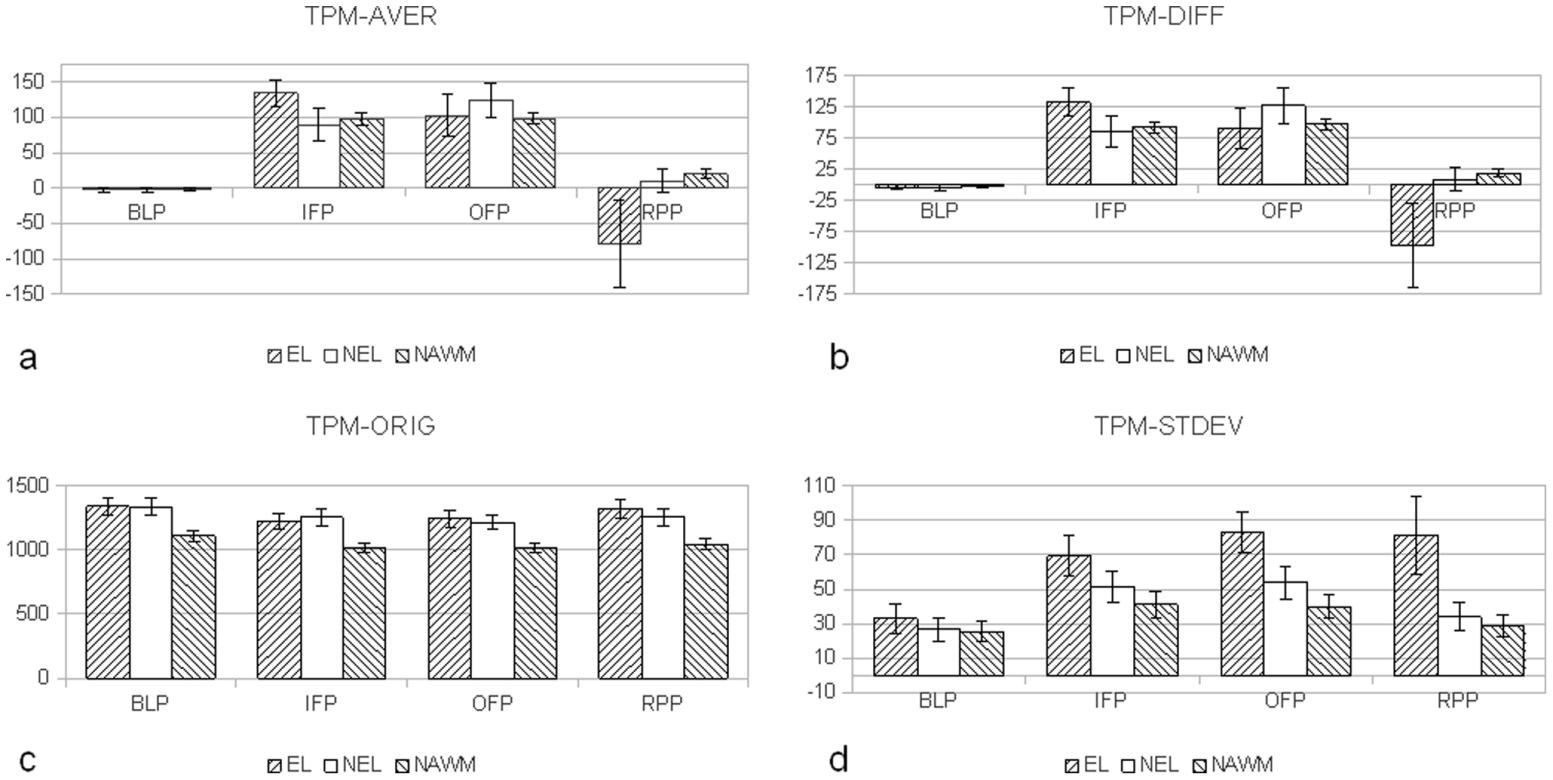
Mean values and 95% confidence intervals for the different TOIs. Data recorded for (a.) TPM-AVER which is a moving averaged version of (b.) TPM-DIFF representing the differences of the baseline images of TPM-ORIG and images recorded during and after bolus passage; (c.) TPM-ORIG representing the original image series, and (d.) TPM-STDEV. The indicated ranges are the 95% confidence intervals.

In Figure 5, 6 the mean values of all 8 TPMs for different time intervals are displayed. The indicated ranges represent the 95% confidence intervals. Differences between ELs, NELs and NAWM can be observed in e.g. TPM-DIFF and TPM-VARI especially during the OFP and RPP phase. Differences between NELs, ELs and NAWMs can be observed in the TPM-ORIG. The differences between the TOIs from TPM-KURT and TPM-SKEW are less pronounced.

**Figure 6:**
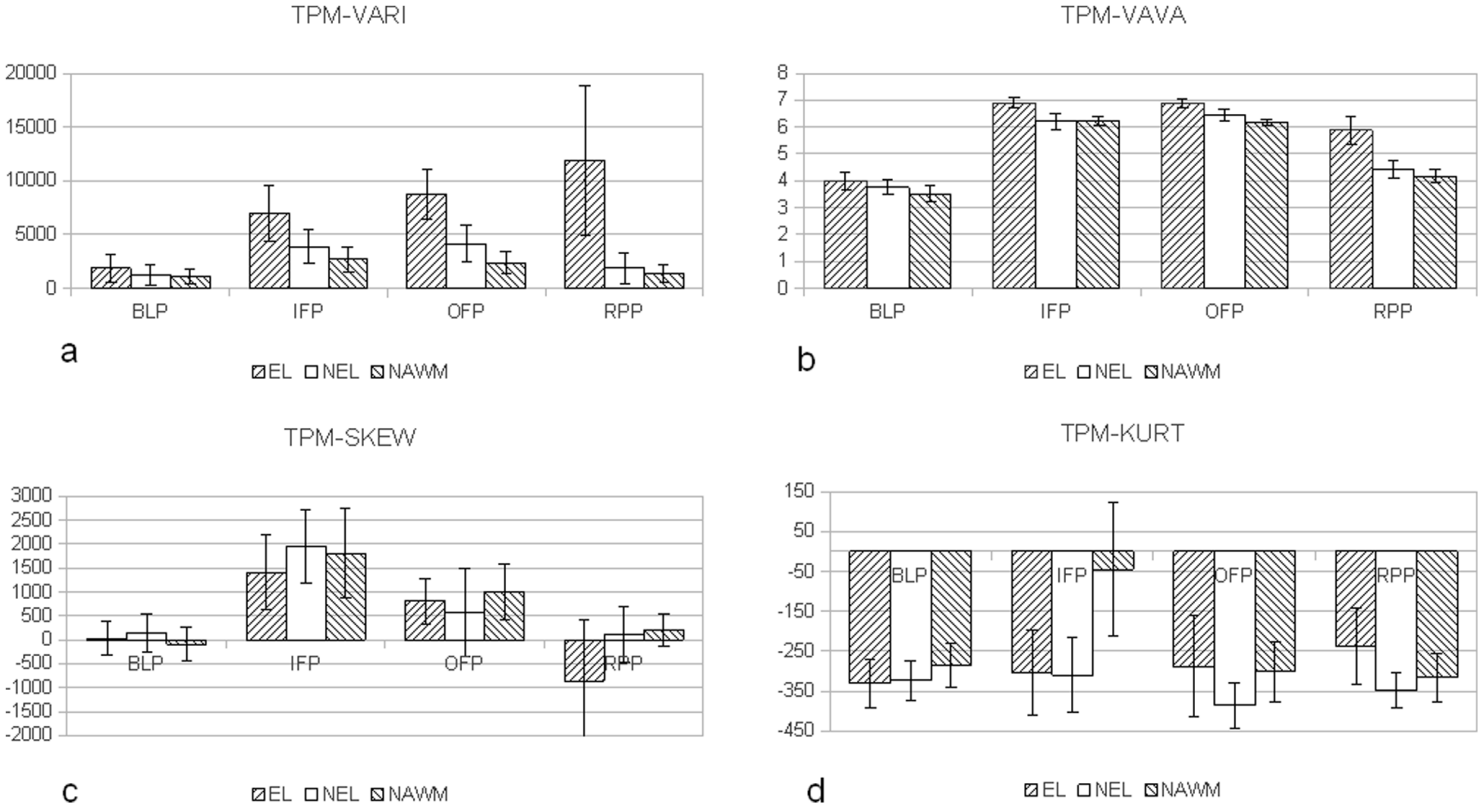
Mean values and 95% confidence intervals for the different TOIs. In (a.) the TPM-VARI results are displayed with monotonically increasing values for NELs; (b.) the TPM-VAVA parameter has a different time behavior that (a.). The mean values of TPM-SKEW (c.) and TPM-KURT (b.) show only little differences for the examined TOIs.

## Discussion

In this paper, we suggest a novel strategy towards in-vivo tissue characterization of ELs, NELs and NAWM in multiple sclerosis. The goal of this pilot study was to investigate the time dependent behavior of statistical texture parameters derived from time series of DSCE difference images. While previous studies aimed at either static texture analysis or description of cerebral blood flow, cerebral blood volume and blood-brain barrier permeability, we aimed to analyze specific texture parameter constellations that differentiate between ELs, NELs and NAWM by analyzing DSCE image time series data. We further analyzed whether these texture parameters evolve as a function of time intervals during bolus passage, and whether these differences are sufficiently large to enable a quantitative grading of the perfusion-related texture of MS lesions. Three points stand out from this study: (a) the voxel intensity distributions of tissue types in the DSCE (difference) image series vary as a function of time during bolus passage; (b) histogram based texture parameters reveal characteristic time responses for ELs, NELs and NAWM; and (c) different tissue types can be discriminated by statistically significant difference in histogram based texture parameter signatures.

The key finding of our study – that DTPA detects subtle differences in time dependent contrast agent distributions otherwise obscured by visual inspection of T1w CE images - implicates that quantitative differences in voxel intensity distributions of the examined DSCE difference images are related to alterations in micro-circulation and BBB properties of the investigated tissue types. The BBB constitutes a dynamic interface forming a neurovascular unit that controls the supply of nutrients while shielding neurons and glial cells off from potentially harmful substances. In MS, GD-DTPA diffuses into the nervous system along the leaky BBB, and conventional MRI provides only a gross estimate of tissue damage. Leukocyte infiltration into the perivascular space is mainly driven by BBB disruption and parenchymal inflammation, although some authors demonstrated passive diffusion of hydrophilic molecules and leukocyte recruitment in post-capillary segments [29]. Local blood flow changes precede the plaque formation process and elude conventional MRI [3]. Inversely, persistence of inflammatory activity along reconstituted BBB in tissue that lacks frank BBB disruption is obscured [30]. The poor clinico-radiological association of the *T*_2_ lesion load in MS may thus be influenced by microscopic inflammation that contributes more strongly to disability [31]. Thus, quantitative knowledge about persistent inflammatory activity would enhance the knowledge about tissue damage beyond a deterministic and analysis of ELs, NELs and NAWM.

Texture analysis applied to MR-images enables such an extraction of *quantitative* information by post-hoc analysis. Different texture parameters have been suggested in the literature: (a) histogram-based parameters; (b) gradient-based parameters; (c) run length-based parameters; (d) co-occurrence matrix-based parameters; (e) auto regressive model-parameters; and (f) wavelet parameters [18]. In the present study, the analysis to *histogram based texture parameters* (i.e. average, variance, skewness, kurtosis and variance-of-the-variance) is restricted to explore whether differences between ELs, NELs and NAWM can be extracted directly from DSCE (difference) images. To the best knowledge of the authors, texture analysis has not been applied previously to *time series* of DSCE-MRI (difference) images aiming to characterize tissue response to a bolus of Gd-containing contrast agent as a function of time.

Previous studies have examined the feasibility of texture analysis to differentiate ELs (88%) and NELs (96%) with high sensitivity [32]. “Coarse” texture analysis in acute enhancing lesions (ELs) predicted tissue injury based on the severity of structural disorganization within acute lesions [33], [34], where “fine” texture refers to a regular pattern and “coarse” texture corresponds to irregular tissue. Texture analysis of 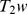 lesions predicted poor recovery and mild ongoing tissue injury [35]. The study further indicated that recovery of acute lesions tends to be associated with the degree of coarse texture during enhancement. While these studies aimed at static characteristics related either to persistence or recovery of acute lesions over time, DTPA analysis first aims at the discrimination of how pathological processes influence the effect of bolus passage of Gadobutrolum on the selected subset of histogram TPs as a function of time and tissue type. Notably, discrimination between ELs, NELs and NAWM was performed on raw EPI images (thus incorporating the *different static and dynamic tissue properties*) during bolus passage and the *difference image* time series (TPM-DIFF) and its derived TPMs. In the latter, the different structural pathological components that influence the static signal components of the tissues are canceled out by the subtraction of the raw image at 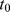. Due to the large differences in the histogram-based TPMs over different time intervals and their tissue dependent specific response, DTPA provides numeric information on a continuous scale about the amount of BBB disruption in MS-lesions, instead of a classification in enhancing or non-enhancing lesions. Yet, a formal proof for a relationship between inflammation and the DTPA information is currently lacking.

Potential relevance of TPMs for the clinical evaluation of MS lesions:

### TPM-ORIG/TPM-DIFF/TPM-AVE

The TPM-ORIG shows monotonously increasing values for ELs during IFP, OFP, and RPP. Yet, this TPM did not discriminate ELs from NELs due to: (a) hypo- and hyper-perfused ELs; and (b) large variations in leakage among ELs; the latter is demonstrated by the TPM-DIFF in Figure 6. The TPM-DIFF further indicates that even NELs continue to have a subtle BBB disruption since the TPM-DIFF is statistically significantly smaller than zero, indicating leakage. This leakage is not observed by visual inspection of 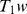 images, but is clearly observed with DTPA. Therefore the TPM-DIFF might be used as a novel gradual marker for BBB disruption that is otherwise discarded on routine imaging. The *difference* in TPM-DIFF (or TPM-AVE) parameter between IFP and RPP (indicated by ΔTPM-DIFF) obtrudes itself as a surrogate marker to grade the MS-lesion state: the higher the value of ΔTPM-DIFF, the larger is the grade of leakage. The ΔTPM-DIFF-value allows grading of the leakage of the MS-lesion on a continuous numeric scale, instead of an ordinal scale known from *T*_1_ *w* imaging (enhancing/non-enhancing). Further clinical studies are necessary to validate the added value ΔTPM-DIFF for the predictive evaluation of disease progression.

### TPM-STDEV/TPM-VAR/TPM-VAVA

These TPMs are all measures of heterogeneity of the examined tissues. For IFP, OFP and RPP the values of the ELs were < NELs < NAWM. TPM-VAR/TPM-STDEV values increase slowly from IFP towards RPP for ELs, and strongly decrease for NELs and NAWM. For TPM-VAVA we observe reductions as a function of time for all three tissue types. A second DTPA-derived numeric MR marker is proposed from the *difference* in the numeric values obtained for the TPM-VAVA between IFP and RPP, and is indicated by ΔTPM-VAVA. The *smaller* ΔTPM-VAVA, the more severely inflammatory is the MS-lesion. This also applies to ΔTPM-STDEV and ΔTPM-VAR. Again, further research must be done to find out whether these parameters correlate with disease activity.

### TPM-SKEW/TPM-KURT

Due to their low SNR (see discussion below) the comparison of the mean values between the different tissues did not reach statistical significance. TPM-SKEW decreased over time in ELs, NELs and NAWM. ELs showed the highest variation in its values including a sign reversal during the late stages. TPM-KURT decreases for NAWM and ELs towards RPP, whereas the ELs increased. For the used 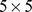 voxel kernel it seems that based on these two texture types no meaningful surrogate markers can be defined. For higher kernel sizes it is likely to find significant differences between these tissue types as well.

### Limitations of the study

**Figure 7:**
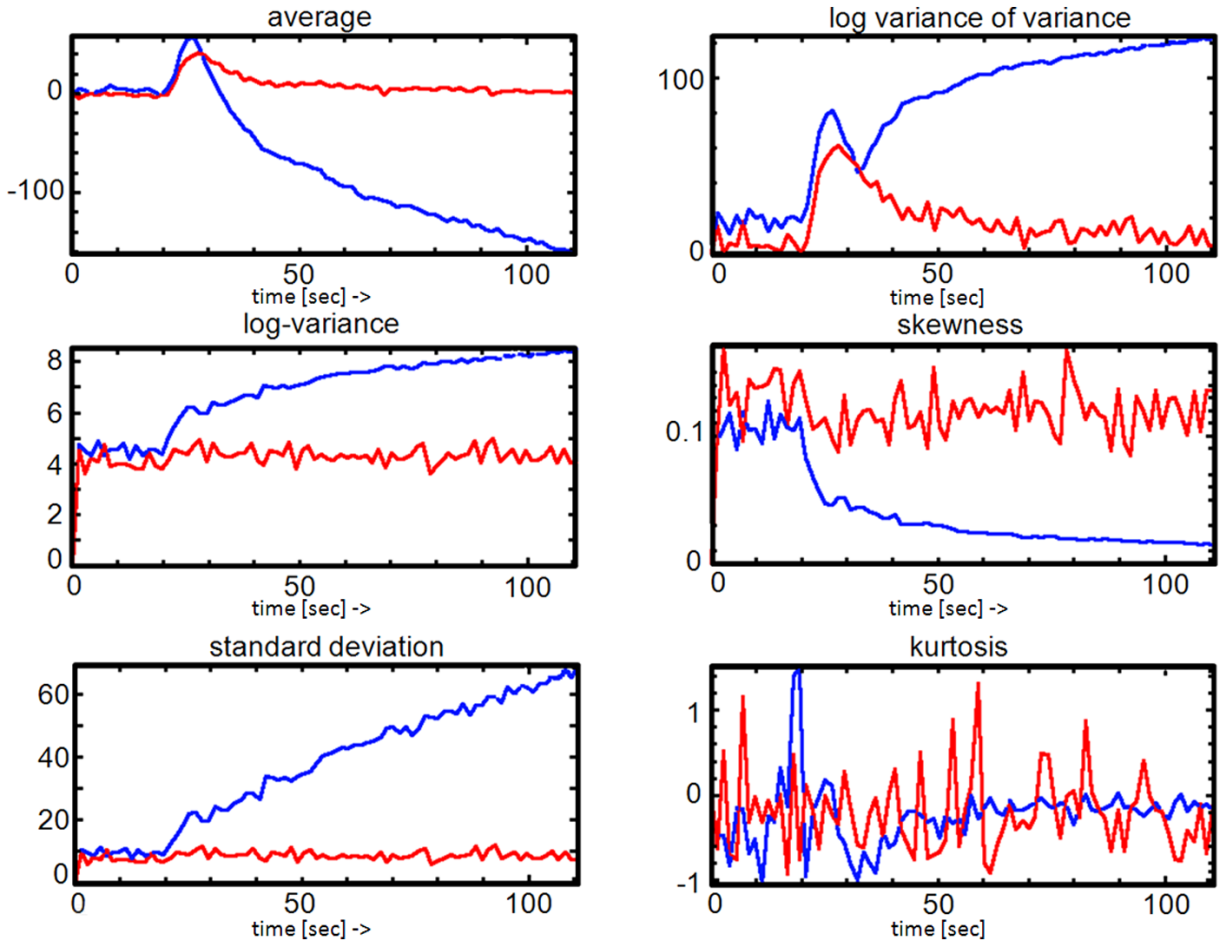
Direct computation and analysis of time dependent texture parameters. The DTPA software also allows direct computation and analysis of time dependent texture parameters without first having to compute the TPMs on a fixed pixel grid. In this figure the time dependent TPs of EL versus NAWM tissue are displayed. Note that each TP has its own SNR.

This study aimed to introduce DTPA and to demonstrate its feasibility in a clinical setting. The female preponderance in our cohort is determined by the epidemiology in MS. The incidence of MS in women is doubling males, and there is still a ongoing disproportional increase [36]. We have investigated different types of lesions in a small and female dominated patient collective diagnosed with different subtypes, which is a drawback. We conclude that large differences in TPs between different tissue types can be detected by DTPA. As a next step, we aim to perform a randomized clinical cohort study in RR-MS to analyze the differences between early and late ELs, NELS and NAWM. SNR of the TPM-SKEW and TPM-KURTs, depends on both SNR of the EPI input images (TPM-ORIG) series *and* kernel size. In this study we choose a kernel size of 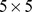 voxels to compute TPMs (see Figure 3 on which the low SNR of the TPM-SKEW and TPM-KURT can be seen). Improved SNR could be obtained by increasing the kernel sizes to be used (e.g. 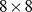), at the cost of spatial resolution however. We identified this SNR/spatial resolution problem during this study and extended the software to allow direct computation of texture parameters from user defined TOIs without the detour via TPMs computed using a fixed kernel. Figure 7 shows the time dependency for an EL and NAWM and using the direct computation method. Note that each TP has its own SNR. Whether the direct computation of texture parameters without first computing the texture parameter maps over a fixed kernel is better than direct computation from the defined TOI is still an open issue and has to be addressed in a separate study.

Finally, this study only examined a limited number of texture parameters and focused on histogram based texture parameters. In a follow up study we will also incorporate gradient-based, cooccurrence-matrix based and run length matrix based TPMs to explore the differences in TPMs of various subtypes of MS lesions.

## Conclusion

This paper investigated the *dynamics* of texture parameter evolution in time series of DSCE-images in 18 patients with CIS or MS according to the 2010 McDonalds criteria. A novel software program is introduced here to investigate time dependent texture parameter maps derived from DSCE (difference) images. In order to enable comparison of texture parameters between two patients, a twofold image-normalization was performed. The first normalization, to compensate for variations in coil-load, is obtained by setting the mean NAWM voxel value during BLP to a specific value in the ORIG-TPM. The second normalization, to compensate for differences in cardiac output, is obtained by normalization of the time integral of the TPM-DIFF over the IFP and OFP for the same NAWM. After normalization, comparisons between patients by comparing the time averaged texture parameter values during the BLP, IFP, OFP and RPP are feasible. We detected dynamic texture features that revealed highly statistically significant differences between ELs, NELs, and NAWM. Based on these dynamic TPs, novel grading parameters for MS lesions can be introduced allowing for grading of MS-lesions on a numerical scale instead of an ordinal scale as is the case with pre/post contrast T1w image analysis. Our data support the hypothesis that, dependent on the tissue type, subtle differences in micro-circulation are present in enhancing and non-enhancing MS lesions.
