# Correction: Characterization of Microcirculation in Multiple Sclerosis Lesions by Dynamic Texture Parameter Analysis (DTPA)

**DOI:** 10.1371/annotation/4f4fb8b4-6730-4134-95f7-6d694f0cfe95

**Published:** 2013-07-30

**Authors:** Rajeev Kumar Verma, Johannes Slotboom, Mirjam Rachel Heldner, Frauke Kellner-Weldon, Raimund Kottke, Christoph Ozdoba, Christian Weisstanner, Christian Philipp Kamm, Roland Wiest

The name of the third author was spelled incorrectly. The correct name is Mirjam Rachel Heldner. 

